# Connectivity Matrix Seriation via Relaxation

**DOI:** 10.1371/journal.pcbi.1011904

**Published:** 2024-02-20

**Authors:** Alexander Borst

**Affiliations:** Max-Planck-Institute for Biological Intelligence, Martinsried, Germany; University of California Santa Barbara, UNITED STATES

## Abstract

Volume electron microscopy together with computer-based image analysis are yielding neural circuit diagrams of ever larger regions of the brain. These datasets are usually represented in a cell-to-cell connectivity matrix and contain important information about prevalent circuit motifs allowing to directly test various theories on the computation in that brain structure. Of particular interest are the detection of cell assemblies and the quantification of feedback, which can profoundly change circuit properties. While the ordering of cells along the rows and columns doesn’t change the connectivity, it can make special connectivity patterns recognizable. For example, ordering the cells along the flow of information, feedback and feedforward connections are segregated above and below the main matrix diagonal, respectively. Different algorithms are used to renumber matrices such as to minimize a given cost function, but either their performance becomes unsatisfying at a given size of the circuit or the CPU time needed to compute them scales in an unfavorable way with increasing number of neurons. Based on previous ideas, I describe an algorithm which is effective in matrix reordering with respect to both its performance as well as to its scaling in computing time. Rather than trying to reorder the matrix in discrete steps, the algorithm transiently relaxes the integer program by assigning a real-valued parameter to each cell describing its location on a continuous axis (‘smooth-index’) and finds the parameter set that minimizes the cost. I find that the smooth-index algorithm outperforms all algorithms I compared it to, including those based on topological sorting.

## Author summary

Connectomic data provide researchers with neural circuit diagrams of ever larger regions of the brain. These datasets are usually represented in a cell-to-cell connectivity matrix and contain important information about prevalent circuit motifs. Such motifs, however, only become visible if the connectivity matrix is reordered appropriately. For example, ordering the cells along the flow of information, feedback and feedforward connections are segregated above and below the main matrix diagonal, respectively. While most previous approaches rely on topological sorting, my method relaxes the discrete vertex indices to real numbers (‘smooth-index’) along independent parameter axes and defines a differentiable cost function, thus, allowing gradient-based algorithms to find a minimum. The parameter set at this minimum is then re-discretized to reorder the connectivity matrix accordingly. I find my method to scale favorably with the circuit size and to outperform all algorithms I compared it to.

## Introduction

Connectomic studies provide neural circuit diagrams of ever larger regions of the brain [1–11]. A natural way to order the elements of such circuit diagrams is along the direction of information flow. This way, import features of the circuit become immediately visible in the respective connectivity matrix: synfire chains [12,13] will form diagonals of the matrix, and squared blocks along the diagonal will indicate the presence of cell assemblies. Arranging the outputs of each neuron within the columns of the connectivity matrix, the ordering will put forward synapses in the lower and recurrent connections in the upper triangle. Isolating recurrent synapses can be achieved by ordering the connectivity matrix such as to push as many entries to the lower triangle as possible, a so-called ‘minimum feedback arc set’. I focus on recurrency as a specific property of neural circuits because of the importance recurrent synapses have for the temporal processing properties of the circuit. In networks without feed-back, the eigenvalues of the corresponding dynamical system matrix *A* = *T*^−1^(*M*−*I*)—with T being the matrix holding the cellular time-constants along the diagonal, M being the connectivity matrix and I the identity matrix—are the negative inverse of the cellular time-constants [14]. In contrast, in networks with feed-back, the eigenvalues depend not only on the cellular time-constants, but in addition on the connectivity parameters [15–17]. Thus, feed-back generally generates new time constants and thereby allows for signal processing at time scales that can go beyond the ones of the isolated elements by orders of magnitude, thereby providing a substrate for working memory [18–20], path integration [21,22] or delay lines in the context of motion vision [23,24]. Within a given connectomic data set, however, neurons usually are not numbered according to the main flow of information within the circuit. In order to retrieve information from the dataset, neurons must therefore be reordered with a specific objective in mind. Reordering of a square matrix with size N x N starts with an index list *π* = (*π*_1_, *π*_2_…*π*_*N*_) which assigns each circuit element i the new index *π*_*i*_. From this, a permutation matrix *P* is created by permuting the columns of the identity matrix accordingly, and the reordered connectivity matrix *R* is obtained by *R* = *PMP*^−1^. Since renumbering preserves the connectivity information, the graphs resulting from renumbering are all isomorphic. Different algorithms exist to reorder matrices [25–33, for review, see 17,34,35]. As a fundamental common property, they all retain the discrete nature of the vertex indices. This way, any property defined to be optimized is not a differentiable function along any parameter axes, but rather a discrete value depending on a given permutation. Since a full enumeration of all permutations grows with O(N!), a brute force approach fails quickly as N increases. The problem presents itself as a sorting problem where each of the different algorithms apply a different, particular strategy.

## Results

**Fig 1:**
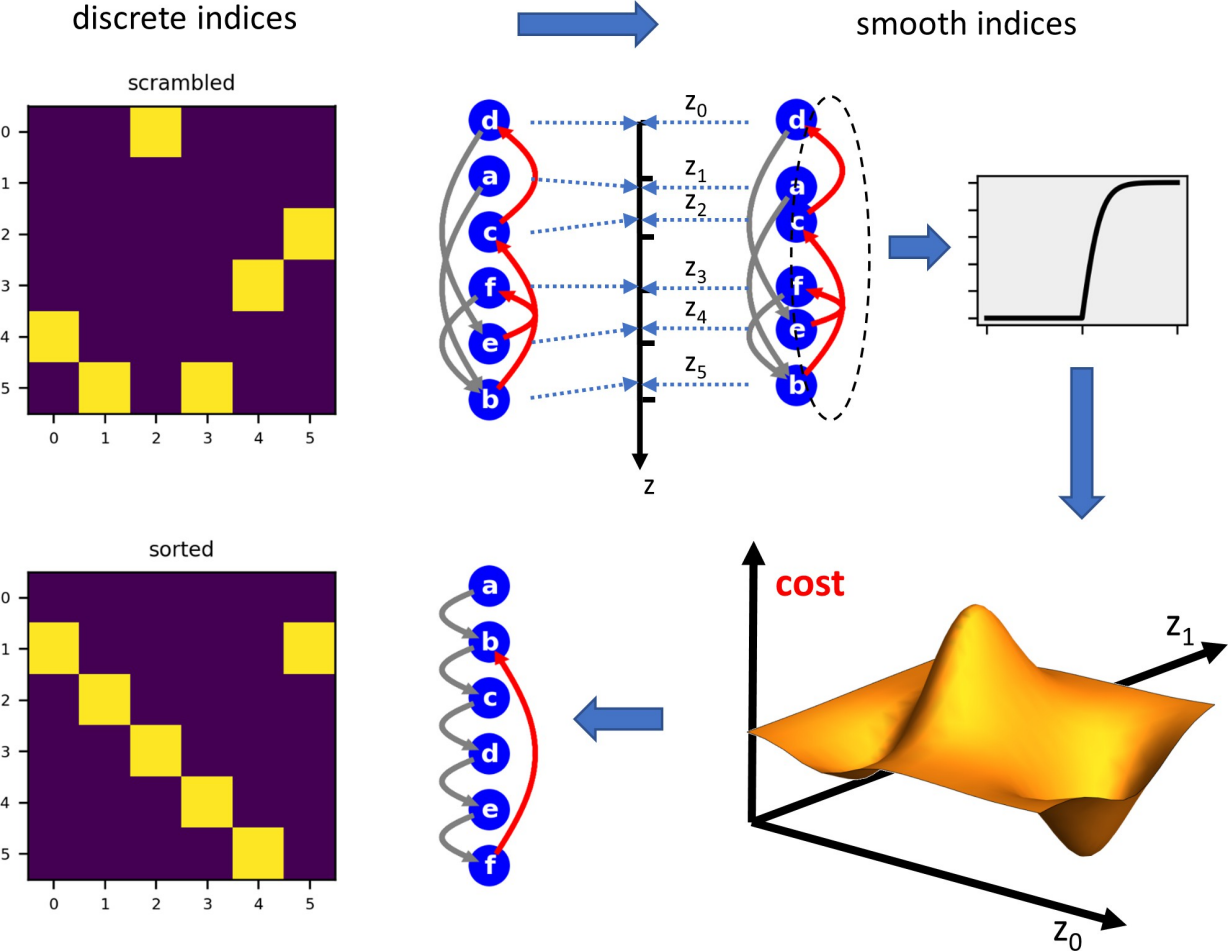
Working scheme of the smooth-index algorithm. Starting from a ‘scrambled’ circuit, the algorithm treats the indices as smooth values along independent parameter axes and minimizes a given cost function, in this case all recurrent connections.

My approach is different from topological searches but rather based on integer relaxation techniques [36–40] (Fig 1). I first relax each vertex index from being a discrete integer number and turn it into a smooth real number z_i_ that indicates the respective vertex position on a continuous axis. Next, each vertex position is considered to be a parameter value along an independent axis. Thus, the cost to be minimized becomes a function of an N-dimensional parameter space. Although the number of possible solutions, i.e. all permutations of N, represent only a small subspace of the new search space N^N^, this transition brings the advantage that the cost function can become differentiable, allowing gradient descent methods to be applied to search for a minimum. A recurrent connection is characterized by the fact that the position z_m_ of a presynaptic neuron m is larger than the position z_n_ of a postsynaptic neuron n. However, the number of recurrent connections varies in a discrete step at the point where z_m_ = z_n_. Hence, it is not a differentiable function of z. I therefore chose the average length z_m_−z_n_ of all recurrent connections as a proxy for the number of recurrent connections, and, by taking a saturating function of the length, such as the logistic function, reduced the stronger influence of longer connections over shorter ones (Eq 1, Methods). To restrict the solutions to the permutation subspace, I require, as an additional criterion to the original cost function, each position to be different from all other positions. To this end, I define an additional ‘Pauli term’ as the mean of the squared differences between the positions and their rank, i.e. their place within the sorted array of positions (Eq 5, Methods). To return to discrete vertex indices, as the final step, the permutation list π that reorders the original matrix is obtained as the vector containing the arguments of the rank-sorted parameters at the minimum of the cost function.

**Fig 2:**
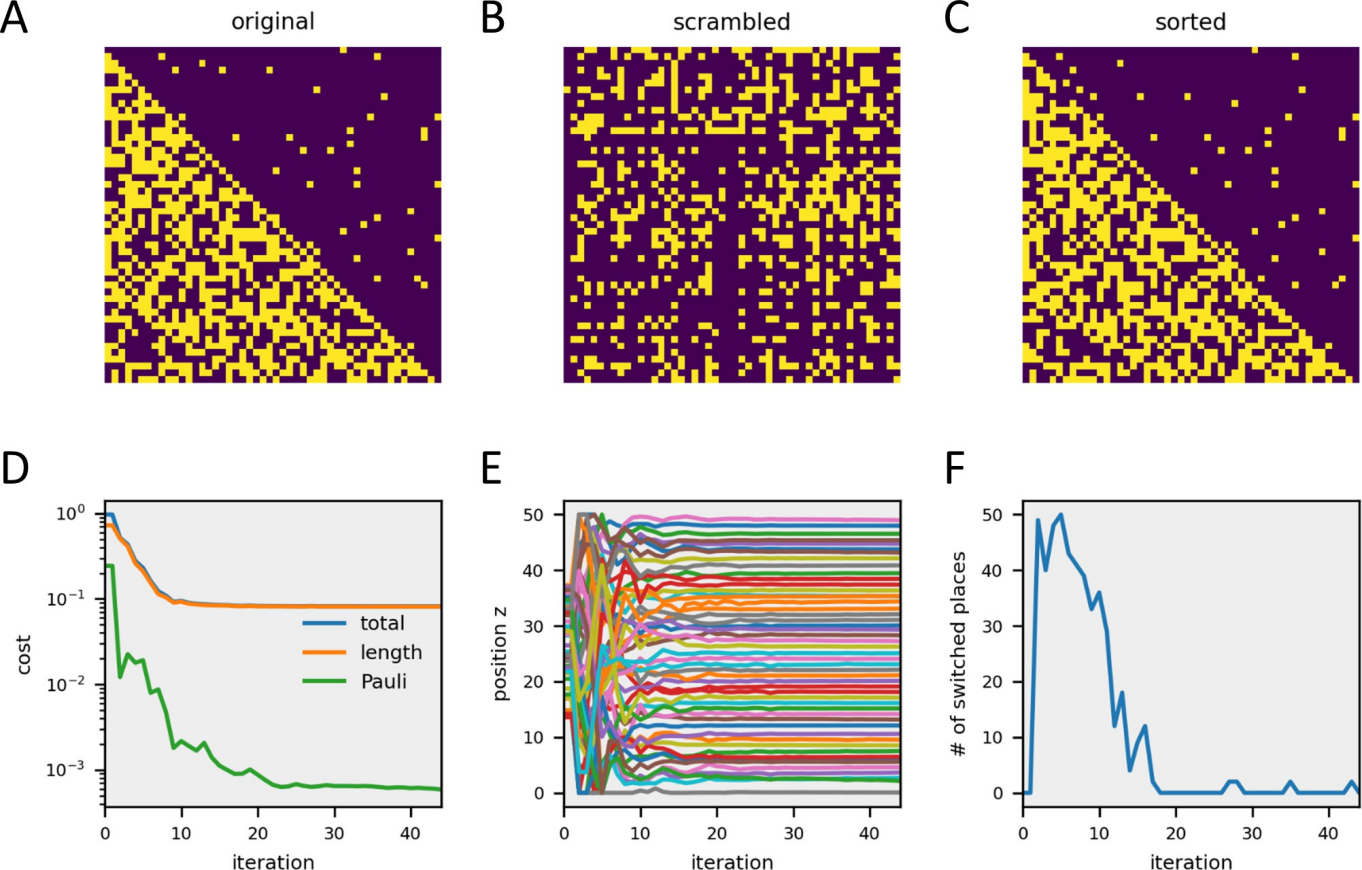
Example performance of the smooth-index algorithm. **A** Original matrix with a few recurrent connections. **B** Scrambled matrix, obtained by random index permutation. **C** Matrix resulting from smooth-index sorting. **D** Different cost functions as the algorithm performs the gradient descent. **E** Positions of all vertices z_i_ during gradient descent. **F** Number of switches, calculated as the number of differences between two consecutive rank-sorted position vectors, during gradient descent.

I implemented the smooth-index algorithm in Python using the Scipy minimize function [41]. For a time-efficient search, I calculated the gradient or ‘Jacobian’ of the cost function (Eq 2 & Eq 6, Methods), which holds all first-order partial derivatives of the cost function along each parameter z_n_. The cost function as well as the Jacobian were passed on to the Scipy minimize function with randomly initialized parameter values z. As shown in Fig 2B–2G for a circuit with 50 neurons, the algorithm works well and isolates a similar number of recurrent synapses as is found in the original connectivity matrix.

**Fig 3:**
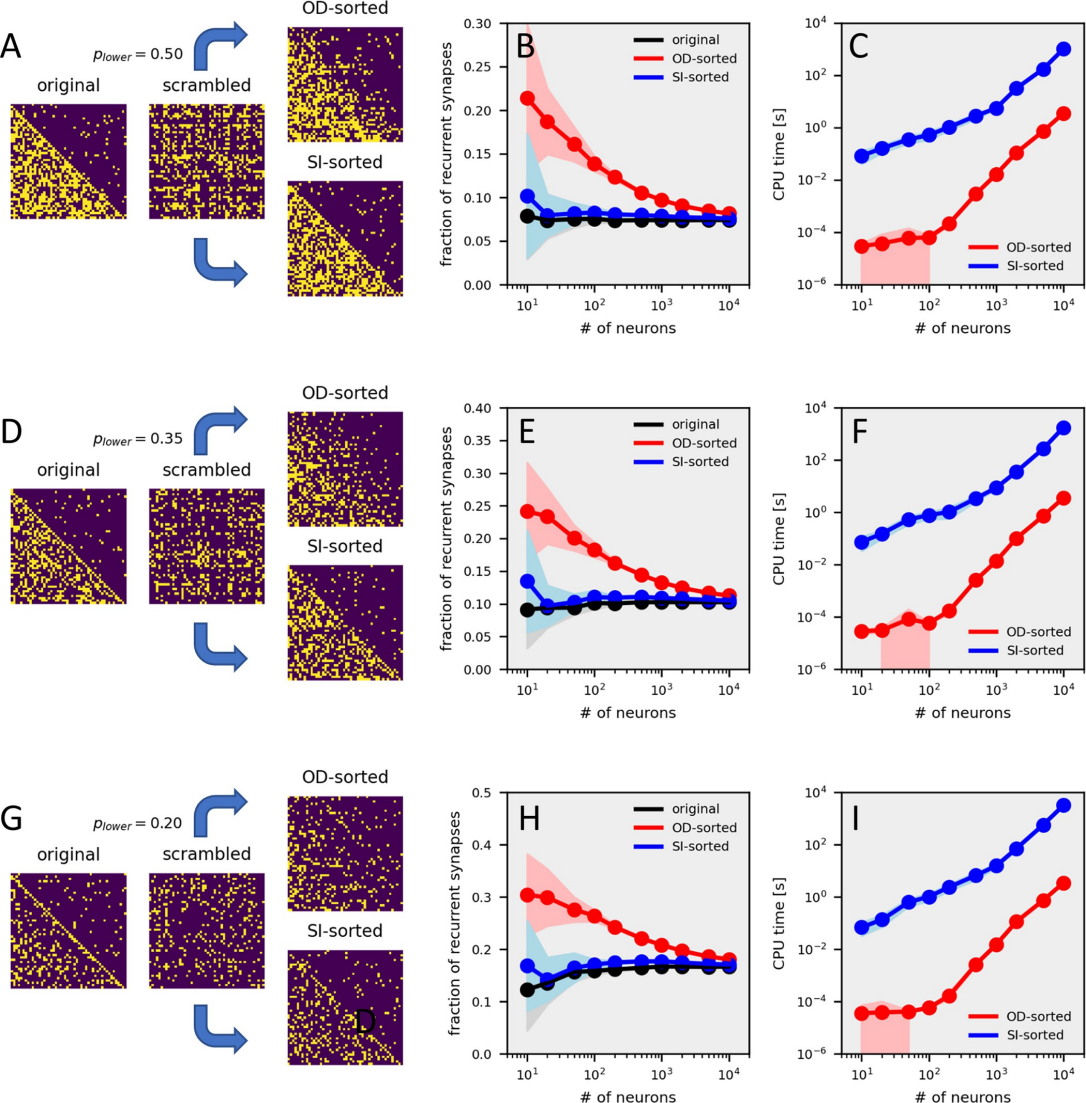
Performance of the smooth-index algorithm for matrices with different densities. **A** Example matrices for N = 50 neurons, given a density of 50% in the lower triangle**. B** Performance of the algorithm as a function of the number of neurons. Plotted is the fraction of non-zero entries in the upper triangle of the matrix in case of the original connectivity matrix before scrambling (in black), after out-degree sorting (in red) and after smooth-index sorting (in blue). **C** CPU time needed for out-degree sorting (in red) and for smooth-index sorting (in blue) as a function of the number of neurons. Data in B and C represent the mean +- standard deviation (shaded area) obtained from 10 sorting runs. **D-F** Same as above, but for a density of 35% in the lower triangle. **G-I** Same as above, but for a density of 20% in the lower triangle.

To test the efficiency of the sorting algorithm quantitatively, I applied it to matrices of different sizes, from 10 up to 10 000 neurons. Each circuit was constructed randomly with a given probability for entries in the lower and upper triangle. To quantify the performance, I calculated the fraction of non-zero entries in the upper triangle of the original connectivity matrix, i.e. before scrambling, and compared that with the fraction after smooth-index sorting and after out-degree sorting. An example of such sorting for a circuit containing 50 neurons is shown in Fig 3A. For circuits with different sizes, the results demonstrate that the algorithm reorders the scrambled connectivity matrices with a high degree of fidelity, in particular for large numbers of neurons (Fig 3B, blue line). Topological sorting according to the out-degree of the nodes performs significantly worse (Fig 3B, red line). For the smooth-index algorithm, the CPU time needed to compute the reordered matrix on a standard desktop computer (Intel i9-7900X CPU with 10 cores, running at 3.3 GHz) grows from about 0.1 seconds for N = 10 to about 1000 seconds for N = 10 000. For larger networks, the CPU time roughly scales with O(N^2^) (Fig 3C, blue line). Not astonishingly, out-degree sorting is always faster (Fig 3C, red line). The desktop was unable to handle networks with more than 20 000 neurons. All performance tests above were applied to matrices with a density of 50% in the lower triangle. In order to see whether smooth-index sorting also performs well with lower density matrices, I ran the same procedure using matrices with a density of 35% (Fig 3D–3F) and 20% (Fig 3G–3I) density in the lower triangle. In all cases, the smooth-index algorithm yields better results than the out-degree sorting, reaching similar values as the original matrix, however with slightly increasing CPU time spent on matrices with lower densities.

**Fig 4:**
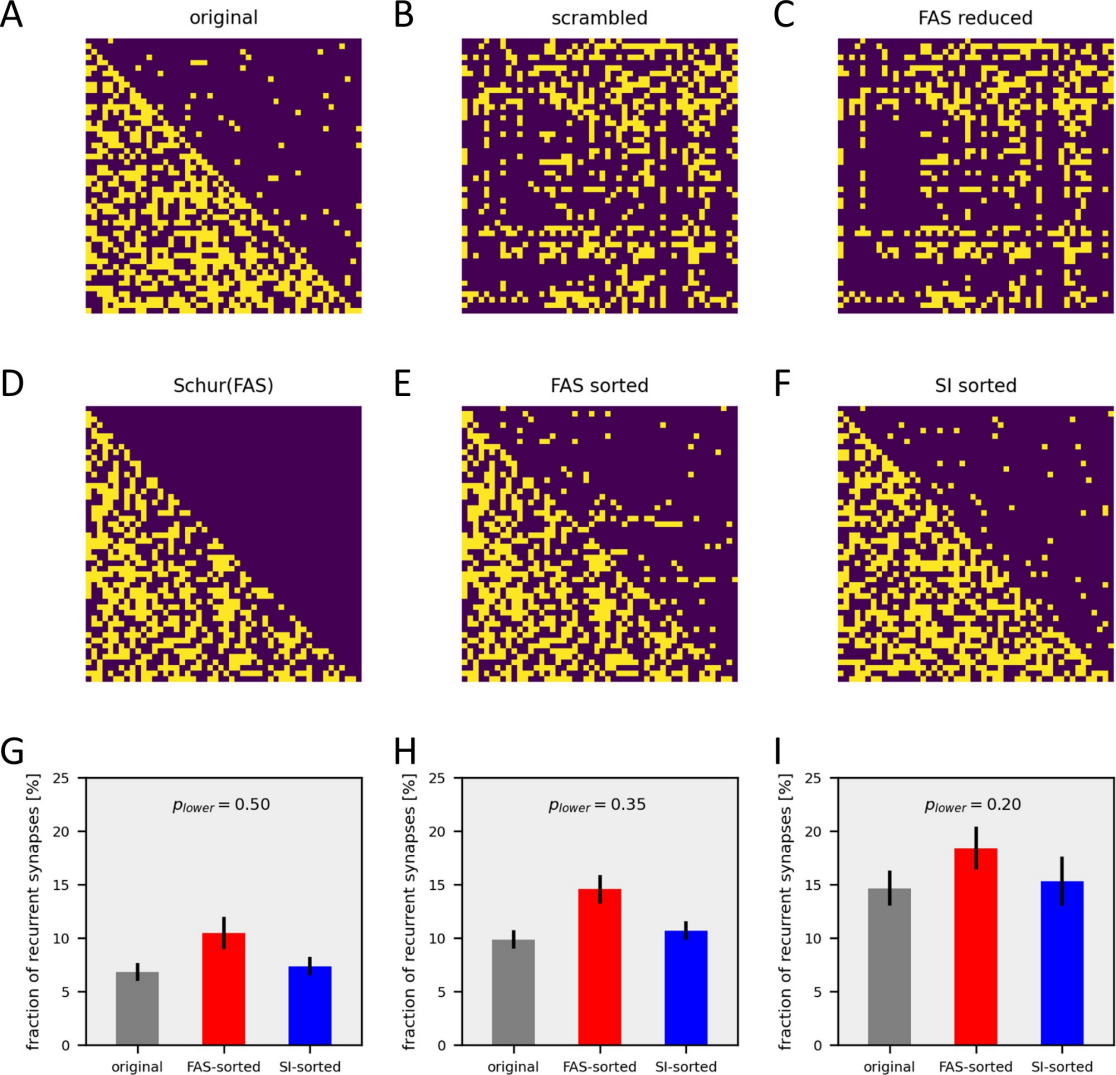
Comparison of the smooth-index (‘SI’) algorithm with feedback arc set (‘FAS’) sorting. **A** Original connectivity matrix of 50 neurons with 50% density in the lower and 4% density in the upper triangle. **B** Same as A, but after scrambling. **C** Resulting matrix after removing recurrent connections identified by the FAS algorithm. Note that the ordering of the scrambled matrix is retained. **D** FAS reduced matrix, reordered applying the Schur decomposition. **E** Full, scrambled matrix reordered applying the permutation matrix as obtained from applying the Schur decomposition to the FAS-reduced matrix. **F** Scrambled matrix after SI-sorting. Note that it has fewer entries in the upper triangle than the one in E. **G-I** Performance of the SI and the FAS algorithm for matrices with three different densities in the lower triangle, from p = 50% to p = 20%. The density in the upper triangle was held constant at 4%. Plotted is the fraction of non-zero entries in the upper triangle of the matrix in case of the original connectivity matrix before scrambling (in gray), after FAS sorting (in red) and after smooth-index sorting (in blue). Data represent the mean +- standard deviation obtained from 10 sorting runs.

I next compared smooth-index sorting with the algorithm developed to identify the minimum feedback arc set (‘FAS’-algorithm) [28]. As a first test, I constructed an original connectivity matrix of 50 neurons with 50% density in the lower and 4% density in the upper triangle (Fig 4A) which was then scrambled randomly (Fig 4B). I then applied the FAS-algorithm to remove the minimum number of recurrent connections identified by the algorithm (Fig 4C). The difference of entries between this matrix and the original one allowed for determining the number of connections identified as being recurrent by the FAS algorithm. However, the remaining elements were still ordered as in the scrambled matrix. For a visual comparison with my algorithm, I then sorted the matrix using the Schur decomposition (Fig 4D). The decomposition uses a transformation matrix *U* (where U*U = *I*, with *I* being the identity matrix), such that *M* = U*RU, where *R* is now lower-triangular. If and only if the circuit is truly feed-forward, what should be the case when the FAS algorithm removes all recurrent connections, the transformation matrix U is a permutation matrix. I then applied the same permutation matrix *U* to sort the scrambled matrix with all its connections intact (Fig 4E) and compared the result with the matrix resulting from SI-sorting of the scrambled matrix (Fig 4F). I applied this procedure to original matrices with three different densities in the lower triangle, i.e. p = 50% (Fig 4G), p = 35% (Fig 4H) and p = 20% (Fig 4I), while the density in the upper triangle was held constant at 4%. Under all three conditions, the FAS algorithm overestimates the number of recurrent connections significantly (compare red and gray bars). In contrast, the SI algorithm results in similar values as found in the original matrix (compare blue and gray bars).

**Fig 5:**
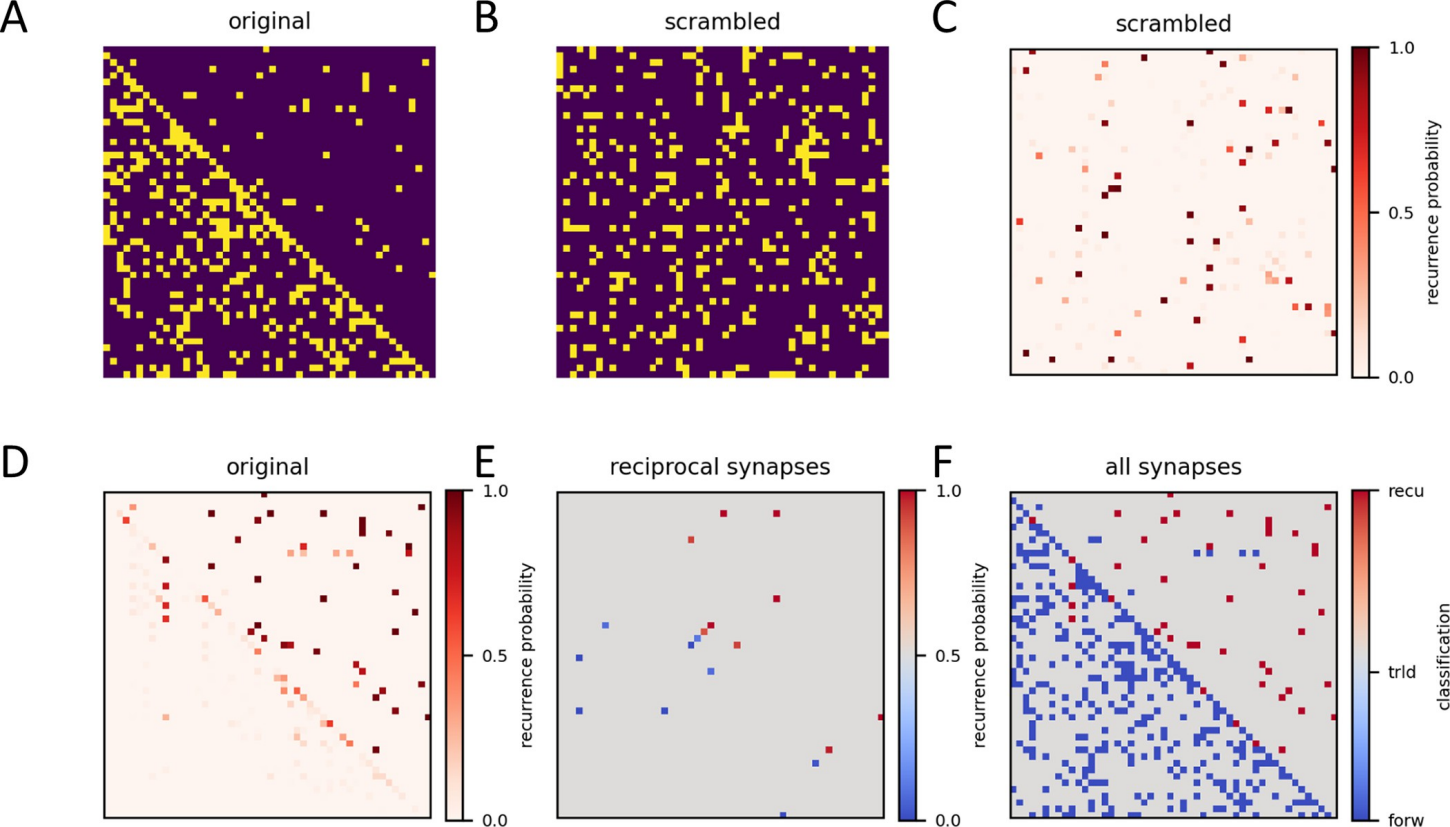
Identification of recurrent synapses by the smooth-index algorithm. **A** Original matrix. **B** Scrambled matrix. **C** Matrix holding the probability of each connection as being classified as recurrent from 1000 sorting runs. **D** Same as C, but after reversed scrambling. **E** Recurrence probability of all reciprocal connections. **F** Original connectivity matrix (in blue) with identified recurrent connections in red (probability threshold = 0.45).

However, yielding a similar number of recurrent connections does not mean that these connections are identical to the ones in the original, unscrambled matrix. In order to see whether I can also identify recurrent synapses, I constructed a single connectivity matrix with about 12% recurrent synapses (Fig 5A), scrambled it once (Fig 5B) and subjected it to the smooth-index sorting 1000 times. With each run being randomly initialized, different minima were likely to be found each time. From all these different runs, I constructed a matrix which holds the probability values by which each connection was classified as recurrent (Fig 5C). Reversing the scrambling revealed that most recurrent synapses of the original matrix were classified as recurrent with a high probability (Fig 5D). Here, an especially interesting case are reciprocal connections between two cells. Given the fact that if one of the connections is feed-forward, the other must be recurrent, one might naively expect that, for reasons of symmetry, each of these reciprocal connections is classified as recurrent with a probability of 0.5. However, contrary to this expectation, looking at all pairs of reciprocal synapses, only one of the connections had a high, while the other had a low probability of being classified as recurrent (Fig 5E). Obviously, taking into account the full connectivity, the symmetry between reciprocally connected neurons breaks. In the end, my method correctly identified 82% of all recurrent connections of the original matrix (Fig 5F). I conclude that my method not only allows for calculating the degree of recurrency, i.e. how many recurrent connections exist in a given network, but also for successfully identifying these recurrent synapses and isolating them from the feed-forward ones.

**Fig 6:**
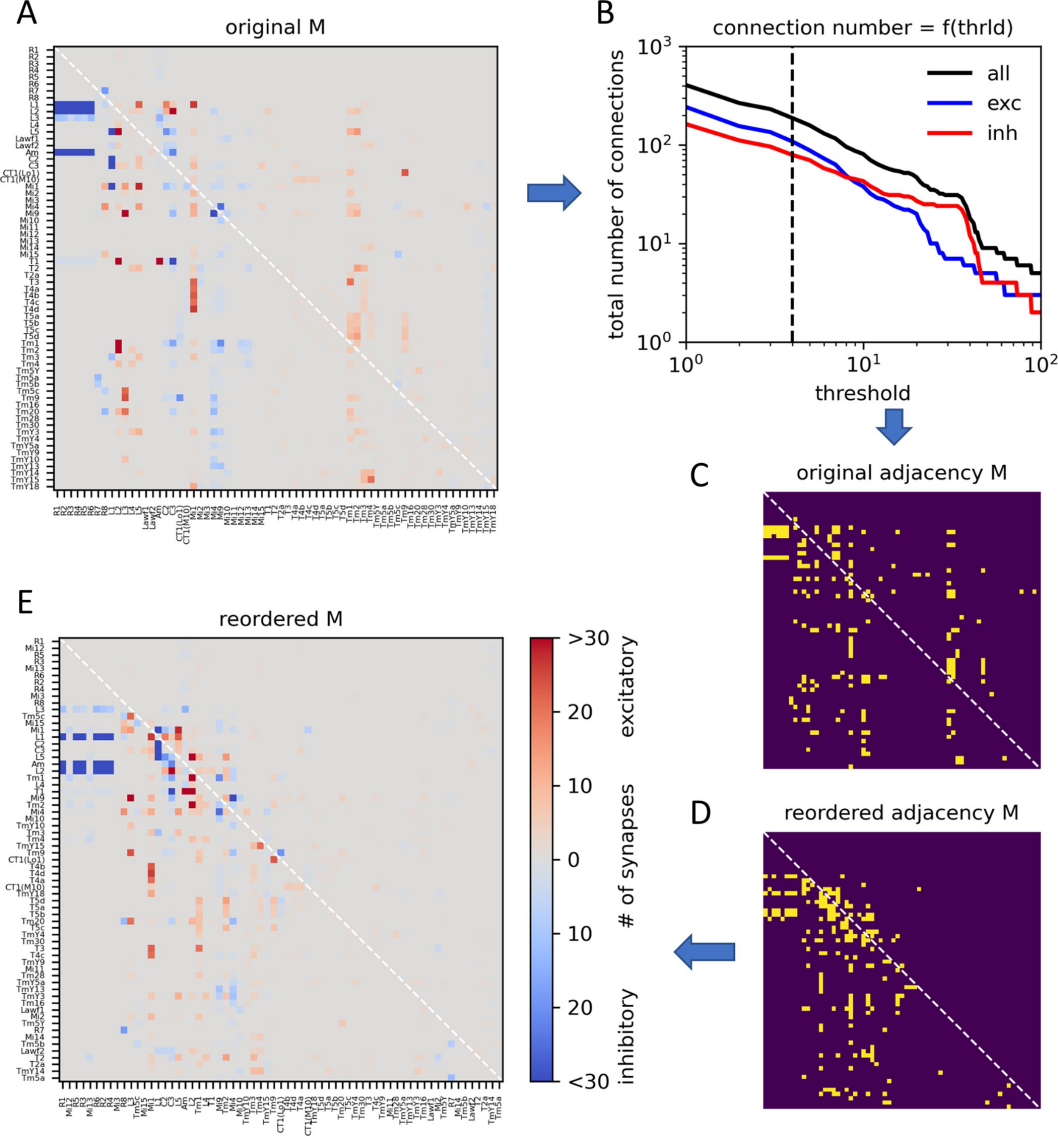
Application of the smooth-index algorithm to the single-column connectome of the Drosophila optic lobe. **A** Connectivity matrix with synaptic weights, ordered according to the sequence of neuropils, from retina to lamina to medulla. **B** Total number of connections as a function of threshold (‘thrld’) for all connections (in black) as well as for excitatory (in blue) and inhibitory (in red) connections, separately. **C** Resulting 0,1-adjacency matrix. **D** Reordered 0,1-adjacency matrix according to the least number of upper triangle entries obtained in 1000 runs of the smooth-index algorithm. **E** Same as D, but shown with the weights of the original weighted matrix.

I next applied my method to a connectome of 65 neurons within a single column of the optic lobe of the fruit fly *Drosophila melanogaster* [9]. This connectome is a weighted connectivity matrix where the non-zero entries indicate the number of synapses found for each connection. Moreover, since neurons can be either excitatory or inhibitory, the sign of the connection is color coded such that excitatory connections are shown in red while inhibitory ones are shown in blue (Fig 6A). I reduced the weighted matrix into a 0,1-adjacency matrix by thresholding the absolute values of the weights at a level of four synapses (Fig 6B and 6C). This matrix was then reordered by the SI-algorithm 1000 times, which was again randomly initialized for each run. From all runs, the one with the lowest number of recurrent connections was chosen to reorder the original adjacency matrix, resulting in a reordered adjacency matrix (Fig 6D). The same reordering was then applied to the original, weighted connectivity matrix, resulting in a weighted, reordered matrix (Fig 6E). The original adjacency matrix had a total of 187 entries, with 67 entries in the upper triangle. 50 of the 187 connections were reciprocal. SI sorting resulted in a matrix with only 30 recurrent connections, i.e. 5 more than the absolute minimum, given 25 pairs of reciprocal connections. Of course, one doesn’t know whether this is indeed the lower limit, but it is safe to state that at least 13% and at most 16% of the connections are recurrent.

In order to examine which connections were classified with a high probability, I determined, how often, in all 1000 runs, each connection was classified as recurrent. The result is shown in Table 1. Contrary to intuition, some connections between lamina neurons and medulla neurons are found to be recurrent with a high probability (row 2: L5 -> Mi1), although anatomy would suggest this to be feed-forward. In order to validate the above findings, I determined all simple paths from Mi1 to L5 and vice versa. Setting a maximum of 2 intermediate nodes, there are 20 paths from Mi1 to L5, but only 6 from L5 to Mi1, confirming the upstream placement of Mi1 with respect to L5. Furthermore, from reciprocally connected neuron pairs like Mi4 and Mi9, the connection from Mi4 to Mi9 is classified with a high probability as recurrent (row22: Mi4 -> Mi9). Again, as in Fig 5, taking into account the full connectivity breaks the symmetry between reciprocally connected neurons breaks defining one direction clearly as feed-forward and the other one as feedback. Five of the connections classified as recurrent are not from reciprocal pairs. These are Tm2 -> L5, Tm1 -> L5, Am -> L3, TmY15 -> Mi4 and Mi9 -> Mi15, highlighted in Table 1.

**Table 1:** List of recurrent connections from the single column connectome of Drosophila. Connections are sorted according to the probability by which a certain connection was classified (1^st^ column, ‘p_recurrency’) as being recurrent in 1000 runs of the smooth-index algorithm. The values in the 2^nd^ column (‘Synaptic Strength’) indicate the number of synapses at each connection, with positive values for excitatory, and inhibitory numbers for inhibitory connections. Highlighted are those connections which are not part of a reciprocally connected pair of neurons.

P_recurrency	Synapse strength	Pre	Post
1.000	4	Tm9	Tm2
0.999	28	L5	Mi1
0.995	4	L2	C3
0.994	8	L5	C2
0.993	-5	Mi4	Tm1
0.988	43	L2	L5
0.982	-19	CT1(Lo1)	Tm9
0.980	7	Tm2	Tm1
0.975	6	TmY10	Mi4
0.973	15	Tm2	L5
0.970	-5	Mi4	L2
0.970	10	Tm1	L5
0.968	-14	Am	L3
0.955	22	L5	L1
0.953	7	Tm2	L2
0.952	5	T1	L2
0.949	6	C3	L1
0.940	-136	L1	Mi1
0.933	-7	CT1(M10)	Mi1
0.833	-5	TmY15	Mi4
0.810	7	L5	C3
0.806	-42	Mi4	Mi9
0.775	5	Tm1	L2
0.763	10	Tm2	Mi9
0.755	-5	Mi9	Mi15
0.728	-20	Mi9	Tm1
0.717	-10	C2	Mi1
0.704	-41	L1	C2
0.698	-6	Mi10	Mi9
0.541	-5	Mi4	Tm2

**Fig 7:**
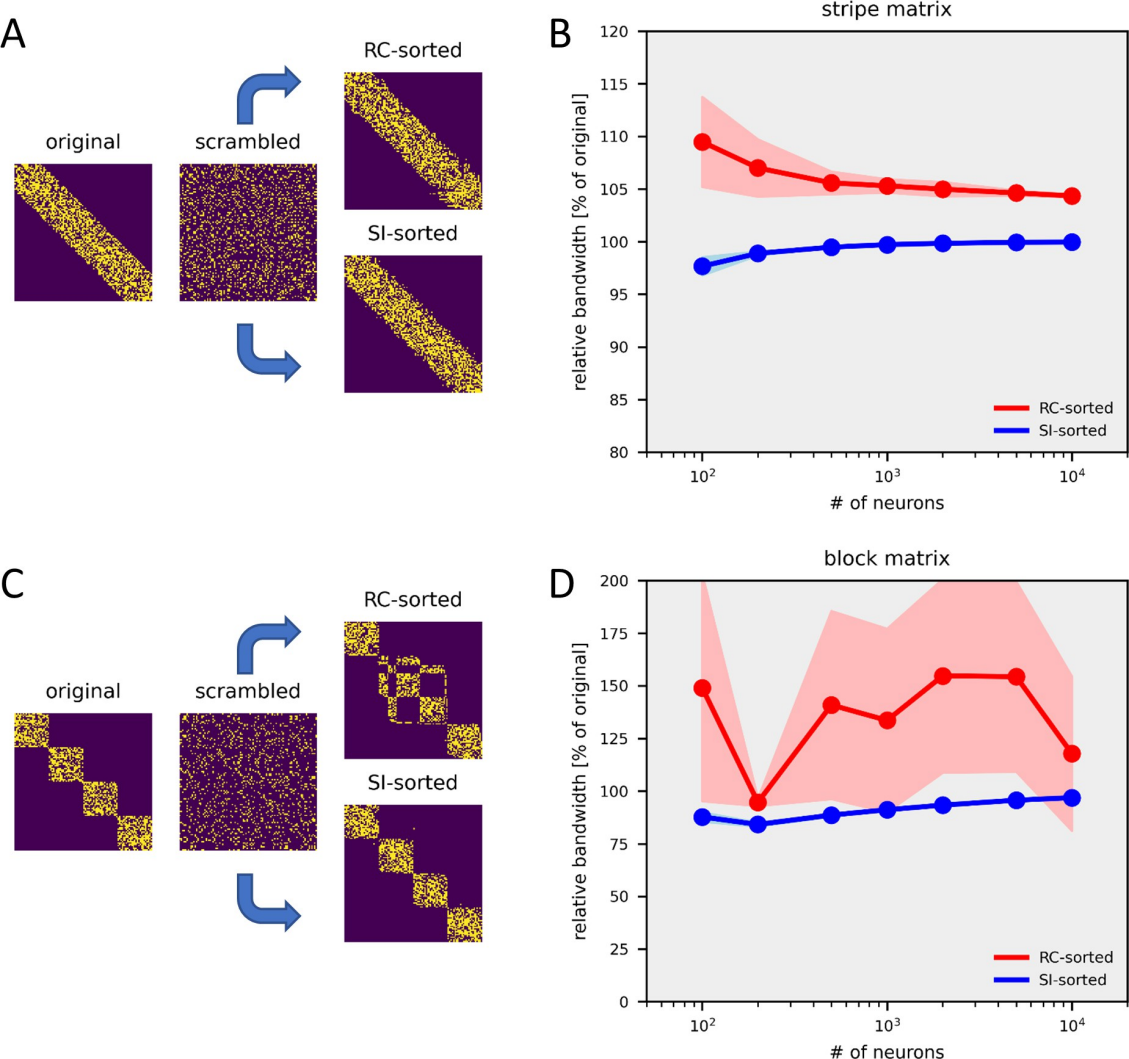
Application of the smooth-index to bandwidth-limited matrices (A,B) and to block-diagonal matrices (C,D). The smooth-index (‘SI’) algorithm is compared with the reverse Cuthill-McKee (‘RC’) algorithm. In A and C, one example sorting is shown for a 50 x 50 matrix. In B and D, the performance of both algorithms is quantified as the bandwidth of the reordered matrix relative to the original matrix. The performance is calculated as the average obtained from 10 runs +- the standard deviation (shaded area).

I finally explored whether the feedback detection problem is unique in being susceptible to smooth-index optimization. Another reordering problem with relevance to Neuroscience is the unmasking of clusters and processing chains. In order to apply the smooth-indexing method to identify properties like a block-diagonal structure or a limited band-width, I define the cost function as the mean squared length of all connections, irrespective of their sign, similar to an earlier approach [38] (Eq 3, Methods). Its gradient is given by Eq 4 (Methods). The algorithm successfully identifies the original structure of a band-width limited connectivity matrix (Fig 7A and 7B) as well as of a block-diagonal connectivity matrix (Fig 7C and 7D) with a high fidelity and outperforms the standard reverse Cuthill-McKee algorithm [25] over circuit sizes up to 10 000 neurons.

## Discussion

Searching for an optimum as a function of permutations within a given range is a hard problem because the number of possible permutations grows with N-factorial. The problem belongs to the large class of problems known as ‘integer programs’ or ‘combinatorial optimization’ which in general are NP-complete [42]. Therefore, various heuristic approaches have been taken such as the FAS algorithm [28–32], the Cuthill-McKee algorithm [25] or page-rank algorithms [33] (for review, see [17,34,35]). Depending on the particular objective and the specific algorithm, they are based on reordering the graph nodes according to their degree, i.e. the number of edges each node has, or partitioning the original graph into subgraphs according to similarity of rows and columns in the respective connectivity matrix. At the end, in one way or the other, these algorithms all test random permutations within the subgraphs in an iterative way, always assessing the reordering according to a given quality criterion. As a common property, all these algorithms keep the vertex indices as discrete values. This is also true for the graph traversal model, applied by Schlegel et al [43] to order the neurons (N ~ 25 000) of the fly olfactory system along the flow of information. In this approach, neurons were grouped into a sequence of functional layers (<10), corresponding to the mean path length from the sensory periphery to any neuron in the graph while taking into account the connection strengths.

Seung [38] was first to apply graph layout techniques [36,37] to reorder matrices. Defining a quadratic cost function for bandwidth minimization, he derived the gradient, set it to 0 and solved the linear matrix equation by using the Moore-Penrose-inversion. While this approach is fast and works reasonably well to identify connection chains and block-diagonal structures, it is, however, not applicable to other, non-quadratic cost functions, like the one needed to isolate recurrent connections. Here, my approach described above is more general and allows for bandwidth minimization as well as for isolation of recurrent synapses, with only a small excess of recurrent connections compared to the original circuit. The approach also differs from Seung [28] by having two cost functions: a data-dependent and a data-independent one. The first one defines the real objective of the minimization, which can be the total length of all connections of the circuit or the number of recurrent connections. The second term is data-independent because it applies to all circuits, whatever their connectivity, but is specific to the method, i.e. to represent the locations of each node as independent axes in a high-dimensional parameter space. In this representation of the problem, besides going from integer to real values for the positions, the space of solutions is only a subspace of the search space: From all combinations of positions, only those are solutions which represent permutations of the numbers from 1 to N. In other words, each single position has to be different from all other ones. This is achieved by the second cost function, called the ‘Pauli-term’.

**Fig 8:**
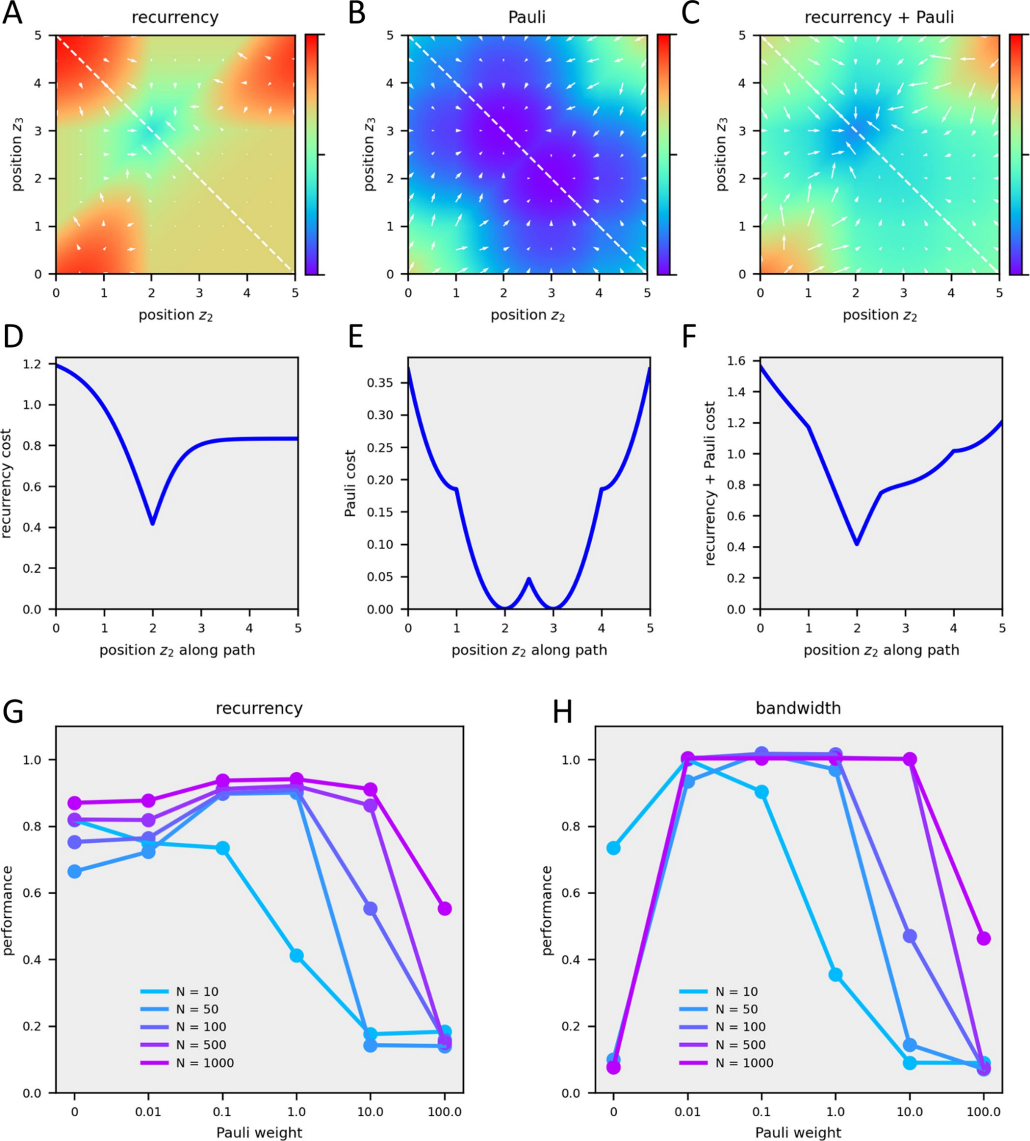
Visualization and balancing the cost function with the Pauli term. **A** Recurrency cost as as f(z_2_,z_3_). **B** Recurrency cost along the dashed line in A. **C** Pauli term as f(z_2_,z_3_). **D** Pauli term along the dashed line in C. **E** Recurrency plus Pauli cost as as f(z_2_,z_3_). **F** Recurrency plus Pauli cost along the dashed line in E. **G** Performance of recurrency minimization as a function of the Pauli term weight. **H** Performance of bandwidth minimization as a function of the Pauli term weight.

To gain some intuition, both cost functions, together with their gradients, are shown as a function of positions z_2_ and z_3_ for the example circuit in Fig 1, with all other positions set to their optimal values, i.e. 0, 1, 4, and 5 (Fig 8A–8F). The recurrency cost is minimal for z_2_ = 2 and z_3_ = 3 (Fig 8A). A 1-dimensional path along the diagonal shows a steep decline towards the minimum and a rise to a plateau thereafter (Fig 8B). The Pauli-cost has two minima, one for z_2_ = 2 and z_3_ = 3, the other for z_2_ = 3 and z_3_ = 2 (Fig 8C). The path along the diagonal shows a steep decline towards the first minimum and steep rise after the second minimum, with a slight energy barrier in between (Fig 8D). In general, the Pauli-cost appears like a bent box of eggs, with small separations between those solutions that represent permutations of positions. The sum of both cost functions again has a single minimum (Fig 8E and 8F), but the plateau in the lower right has a higher gradient towards the minimum than the recurrency term alone.

It is important to note that the Pauli-term only alleviates finding the optimum ordering of the connectivity matrix: in itself, it does not contribute to the quality of the result since taking the arguments of the rank-sorted positions guarantees each position to be an integer value and to be different from any other one anyway. For this reason, also, the optimal balance between the two terms of the cost function cannot be found by treating the relative weight of the Pauli-term as an additional parameter in the gradient descent since it will always lead to zero weight, or the value set by the boundary condition of the parameter, respectively. I, therefore, set the relative weights of the two parts of the cost function empirically. As is shown in Fig 8G and 8H), the relative weight can vary over a broad range.

For an application of the smooth-index algorithm to real connectomes, it might be desirable to include the synaptic weights at each entry of the connectivity matrix. This can be achieved in two different ways. Most easily, the strength of connections can be preserved by sorting the binary connectivity matrix and applying the resulting index list to permutate the original connectivity matrix (Fig 6). Alternatively, the synaptic weights of the original connectivity matrix can enter the cost function by giving stronger connections more weight, this way affecting the sorting outcome itself. Future work will show to what extent my approach is generalizable to other problems in the field of matrix reordering as well.

## Material and methods

### 1. Formulas of cost functions and their gradients

In the following, z_m_ and z_n_ denote the positions z of a neuron m presynaptic to a neuron n, respectively.

I define Δ*z*_*n*,*m*_ as: Δ*z*_*n*,*m*_ = *z*_*n*_−*z*_*m*_+1

The Heaviside function H(x) as: $H(x)=\left\{ \begin{array}{c} 1\;if\;x\geq 0\\ 0\;if\;x<0 \end{array}\right.$

The logistic function *α*(*x*, *N*) as: $\alpha (x)=\frac{1}{1+e^{-10x/N}}$

To minimize recurrent connections, I define the length term *L*(*z*) as:

$$L(z)=\frac{1}{\sum M}\sum_{n\in N}\sum_{m\in N}M_{n,m}[\alpha (\Delta z_{m,n})-\frac{1}{2}]H(\Delta z_{m,n})$$ (1)

Using $\alpha^{\prime }(x)=\alpha (x)(1-\alpha (x))$, the Jacobian of *L*(*z*) becomes:

$$\frac{\partial L(z)}{\partial z_{n}}=\frac{10}{N}[-\sum_{k\in N}M_{n,k}\alpha^{\prime }(\Delta z_{k,n})H(\Delta z_{k,n})+\sum_{k\in N}M_{k,n}\alpha^{\prime }(\Delta z_{n,k})H(\Delta z_{n,k})]$$ (2)

To minimize the bandwidth, I define the length term *L*(*z*) as:

$$L(z)=\frac{1}{N^{2}}\frac{1}{\sum M}\sum_{n\in N}\sum_{m\in N}M_{n,m}{\left(\Delta z_{m,n}\right)}^{2}$$ (3)

The Jacobian of *L*(*z*) becomes:

$$\frac{\partial L(z)}{\partial z_{n}}=\frac{1}{N^{2}}\frac{2}{\sum M}[-\sum_{k\in N}M_{n,k}\Delta z_{k,n}+\sum_{k\in N}M_{k,n}\Delta z_{n,k}]$$ (4)

In addition, I define the Pauli term *P*(*z*) as:

$$P(z)=\frac{1}{N^{3}}\sum_{n\in N}{\left(z_{n}-rank(z_{n})\right)}^{2}$$ (5)

The Jacobian of *P*(*z*) becomes:

$$\frac{\partial P(z)}{\partial z_{n}}=\frac{2}{N^{3}}(z_{n}-rank(z_{n}))$$ (6)

The total cost function *C*(*z*) is defined as: *C*(*z*) = *L*(*z*)+*P*(*z*)

### 2. Continuity and differentiability of the Pauli term

In those regions where the positions remain in the same order, i.e. their ranks do not change, the Pauli function is the sum of quadratic functions of the positions, offset by their respective ranks. It is therefore sufficient to analyze continuity and differentiability at the switching points, i.e. where the change of one position z_i_ leads to a change of the rank of z_i_ as well as of its neighbor z_j_. All other components of the function (denoted as K) remain unaltered. I denote the rank(z_i_) as r, the rank of its larger neighbor z_j_ then becomes r+1. Note that after switching, i.e. when z_i_ > z_j_, the rank of z_i_ becomes r+1, the one of z_j_ becomes r. In the following,

$P(z)=\frac{1}{N^{3}}\sum_{n\in N}{\left(z_{n}-rank(z_{n})\right)}^{2}$ simplifies to:

$$P(z)={\left(z_{i}-r\right)}^{2}+{\left(z_{j}-(r+1)\right)}^{2}+K$$

Continuity:

Left approach:

$$\underset{\varepsilon \to 0}{\lim}P(z_{i}=z_{j}-\epsilon )=\underset{\varepsilon \to 0}{\lim}({\left(z_{j}-\epsilon -r\right)}^{2}+{\left(z_{j}-(r+1)\right)}^{2}+K)=$$

$$\underset{\varepsilon \to 0}{\lim}({\left(z_{j}-r-\epsilon \right)}^{2}+{\left(z_{j}-r-1\right)}^{2}+K)={2(z_{j}-r)}^{2}-2(z_{j}-r)+1+K$$

Right approach:

$$\underset{\varepsilon \to 0}{\lim}P(z_{i}=z_{j}+\epsilon )=\underset{\varepsilon \to 0}{\lim}({\left(z_{j}+\epsilon -(r+1)\right)}^{2}+{\left(z_{j}-r\right)}^{2}+K)=$$

$$\underset{\varepsilon \to 0}{\lim}({\left(z_{j}-r+\epsilon -1\right)}^{2}+{\left(z_{j}-r\right)}^{2}+K)={2(z_{j}-r)}^{2}-2(z_{j}-r)+1+K$$

Since left and right approach leads to the same value, the Pauli function is continuous at the switching points.

Differentiability:

Left approach:

$$\underset{\varepsilon \to 0}{\lim}\frac{\partial P(z_{i}=z_{j}-\epsilon )}{\partial z_{i}}=\underset{\varepsilon \to 0}{\lim}2(z_{j}-\epsilon -r)=2(z_{j}-r)$$

Right approach:

$$\underset{\varepsilon \to 0}{\lim}\frac{\partial P(z_{i}=z_{j}+\epsilon )}{\partial z_{i}}=\underset{\varepsilon \to 0}{\lim}2(z_{j}+\epsilon -(r+1))=2(z_{j}-r)-2$$

Since left and right approach leads to different values, the Pauli function is not differentiable at the switching points.

### 3. Matrix generation

Original matrices, i.e. before scrambling, for the data shown in Fig 2 were constructed in the following way: elements in the lower triangle were set to 1 with a probability = 0.5, in the upper triangle with a probability = 0.04, elements along the first subdiagonal with a probability = 1. For the data in Fig 3, stripe and block matrices were constructed such that each element within the stripe or block was set to 1 with a probability = 0.5. The stripe width was set to be 40%, the block size was set to be 25% of the matrix size N.

### 4. Summary description of the smooth-index algorithm

I first turn each vertex index from a discrete integer number into a smooth real number z_i_ indicating the respective vertex position on a one-dimensional axis. Next, each vertex position is considered to be a parameter value along an independent axis. Thus, the cost becomes a function of an N-dimensional parameter space. This transition brings the advantage that the cost function can become differentiable, allowing gradient descent methods to be applied to search for a minimum. I applied this idea to reorder matrices such as a) to isolate recurrent connections, and b) to minimize the bandwidth.

a) Recurrency minimization: A recurrent connection is characterized by the fact that the position z_m_ of a presynaptic neuron m is larger than the position z_n_ of a postsynaptic neuron n. However, the number of recurrent connections varies in a discrete step at the point where z_m_ = z_n_, and, hence, is not a differentiable function of z. I therefore chose the average length z_m_−z_n_ of all recurrent connections as a proxy for the number of recurrent connections. Taking a saturating function of the length reduced the stronger influence of longer connections over shorter ones. The cost function and its gradient are formulated by Eq 1 and Eq 2, respectively.

b) Bandwidth minimization: Here, the cost is defined as the mean of the squared length of all connections, irrespective of their sign. The respective cost function and its gradient are formulated by Eq 3 and Eq 4, respectively.

c) The Pauli term: To keep all positions from collapsing into a single value, I define an additional Pauli term as the mean of the squared differences between the positions and their rank within the sorted position array. This part of the cost function and its gradient are formulated by Eq 5 and Eq 6, respectively.

The total cost is defined as the sum of the specific cost function and the Pauli term.

Both the total cost function as well as its gradient (Eqs 1,2,5,6 for minimizing recurrent connections, Eqs 3,4,5,6 for minimizing bandwidth) are passed onto the Python Scipy minimize function [41] using the bound constrained method of Broyden, Fletcher, Goldfarb, and Shanno (’L-BFGS-B’). To return to discrete vertex indices, as the final step, the permutation list π that reorders the matrix is obtained as the vector containing the arguments of the rank-sorted parameters at the minimum of the cost function. Termination of the search is set by the tolerance value used by the Scipy minimize function. This value is set in the ‘SI_sort’ function of the library to different values depending on the size of the circuit. The default value is 10^−8^ and decreases for larger circuits down to 10^−11^.
